# Initial evaluation of (4*S*)-4-(3-[^18^F]fluoropropyl)-l-glutamate (FSPG) PET/CT imaging in patients with head and neck cancer, colorectal cancer, or non-Hodgkin lymphoma

**DOI:** 10.1186/s13550-020-00678-2

**Published:** 2020-08-28

**Authors:** Sonya Y. Park, Camila Mosci, Meena Kumar, Mirwais Wardak, Norman Koglin, Santiago Bullich, Andre Mueller, Mathias Berndt, Andrew W. Stephens, Frederick T. Chin, Sanjiv S. Gambhir, Erik S. Mittra

**Affiliations:** 1 Department of Radiology, Division of Nuclear Medicine, Seoul St. Mary’s Hospital, College of Medicine, The Catholic University of Korea, Seoul, Republic of Korea; 2 Molecular Imaging Program at Stanford (MIPS), Department of Radiology, Stanford University School of Medicine, Stanford, CA USA; 3 Bayer Pharma AG, Berlin, Germany; 4 Life Molecular Imaging GmbH, Berlin, Germany; 5 Department of Bioengineering, Stanford University, Stanford, CA USA; 6 Department of Materials Science & Engineering, Stanford University, Stanford, CA USA; 7 Bio-X Program, Stanford University, Stanford, CA USA; 8 Department of Diagnostic Radiology, Division of Nuclear Medicine & Molecular Imaging, Oregon Health & Science University, 3181 SW Sam Jackson Park Rd., Mail Code L340, Portland, OR 97239 USA

**Keywords:** Glutamate, x_C_^−^ transporter, [^18^F]FSPG, Positron emission tomography, PET/CT, Head and neck cancer, Colorectal cancer, Lymphoma

## Abstract

**Purpose:**

(4*S*)-4-(3-[^18^F]Fluoropropyl)-l-glutamic acid ([^18^F]FSPG) measures system x_C_^−^ transporter activity and shows promise for oncologic imaging. We present data on tumor uptake of this radiopharmaceutical in human subjects with head and neck cancer (HNC), colorectal cancer (CRC), and non-Hodgkin lymphoma (NHL).

**Methods:**

A total of 15 subjects with HNC (*n* = 5), CRC (*n* = 5), or NHL (*n* = 5) were recruited (mean age 66.2 years, range 44–87 years). 301.4 ± 28.1 MBq (8.1 ± 0.8 mCi) of [^18^F]FSPG was given intravenously to each subject, and 3 PET/CT scans were obtained 0–2 h post-injection. All subjects also had a positive [^18^F]FDG PET/CT scan within 1 month prior to the [^18^F]FSPG PET scan. Semi-quantitative and visual comparisons of the [^18^F]FSPG and [^18^F]FDG scans were performed.

**Results:**

[^18^F]FSPG showed strong uptake in all but one HNC subject. The lack of surrounding brain uptake facilitated tumor delineation in the HNC patients. [^18^F]FSPG also showed tumor uptake in all CRC subjects, but variable uptake in the NHL subjects. While the absolute [^18^F]FDG SUV values were comparable or higher than [^18^F]FSPG, the tumor-to-background SUV ratios were greater with [^18^F]FSPG than [^18^F]FDG.

**Conclusions:**

[^18^F]FSPG PET/CT showed promising results across 15 subjects with 3 different cancer types. Concordant visualization was mostly observed between [^18^F]FSPG and [^18^F]FDG PET/CT images, with some inter- and intra-individual uptake variability potentially reflecting differences in tumor biology. The tumor-to-background ratios were greater with [^18^F]FSPG than [^18^F]FDG in the cancer types evaluated. Future studies based on larger numbers of subjects and those with a wider array of primary and recurrent or metastatic tumors are planned to further evaluate the utility of this novel tracer.

## Introduction

Tumor cells require an adequate supply of nutrients to meet anabolic and energetic needs while maintaining appropriate redox balance for growth, proliferation, and survival. Recent research revealed several interesting insights into tumor metabolism, including how tumor cells adopt metabolic pathways to cope with such demands in challenging environments [1, 2]. Whereas the upregulation of the glycolytic pathways has long been described and [^18^F]FDG PET is widely used in clinical routine for tumor imaging, other dominant metabolic pathways in tumors such as the glutaminolytic pathway or glutathione biosynthesis/redox-balancing pathway are now being explored in more detail. The glutaminolytic pathway provides both energy and building blocks for tumor growth [3] and can be investigated by PET probes targeting the alanine serine cysteine-preferring transporter 2 (ASCT2 or SLC1A5) [4]. The glutathione biosynthesis/redox-balancing pathway can be investigated by targeting the system x_C_^−^ transporter [5]. This antiporter consist of two subunits, SLC7A11 (also known as xCT subunit), which is the catalytic subunit that mediates transport function, and SLC3A2 (also known as 4F2hc or CD98hc subunit), which functions as a chaperone and recruits the SLC7A11 subunit to the plasma membrane. Extracellular cystine gets imported in exchange for efflux of intracellular glutamate. Cystine is then reduced intracellularly to two molecules of cysteine. The sulfur-containing cysteine molecules can be incorporated into proteins or used in the biosynthesis of the antioxidant agent glutathione. Improved access to glutathione provides advantages for tumor cell survival by allowing better detoxification of chemotherapeutics and reactive oxygen species while inhibition of system x_C_^−^ has been shown to induce tumor-selective ferroptosis and suppresses tumor growth in various tumor models [6, 7].

[^18^F]FDG remains the primary radiotracer used clinically in the evaluation of various cancers [8–10]. However, [^18^F]FDG also has its known limitations. Prominent background uptake in the brain, kidneys, and often the gastrointestinal tract can reduce the sensitivity of [^18^F]FDG in those regions. Another challenge is the prominent [^18^F]FDG uptake seen in benign, inflammatory lesions such as infections, granulomatous processes, and sarcoidosis [11–13]. This can reduce the specificity of [^18^F]FDG for malignancy and make the interpretation of certain scans difficult, demanding the development of other tumor imaging probes. [^18^F]FDG PET uptake shows correlation with many parameters such as tumor aggressiveness, proliferative activity, and prognosis [14, 15] but does not necessarily inform about other tumor characteristics. These limitations stimulated the development of new PET tracers to study other important aspects of tumor metabolism.

For example, (4*S*)-4-(3-[^18^F]fluoropropyl)-l-glutamate (previously BAY 94-9392 and herein referred to as [^18^F]FSPG) is an investigational ^18^F-labeled glutamate derivative for PET imaging of system x_C_^−^ transporter activity. Specific transport of [^18^F]FSPG via the x_C_^−^ transporter was demonstrated in cell competition assays and xCT knock-down cells, and excellent tumor visualization was achieved in animal tumor models [16]. Biodistribution analysis in rodents showed rapid blood clearance via the kidneys and low background activity from healthy tissue, providing high contrast for tumor imaging. In cancer models, [^18^F]FSPG demonstrated the ability to identify drug-resistance by detecting upregulated antioxidant pathways and provides an early redox indicator of tumor response to treatment, preceding other markers such as tumor shrinkage and decreased glucose utilization [17, 18]. [^18^F]FSPG did not accumulate in the inflammatory model tested in animals [16], although subsequent clinical studies reported uptake in sarcoidosis [19]. Pilot clinical studies examining dosimetry and biodistribution in healthy volunteers [20, 21] and tumor detection in patients with non-small cell lung cancer, hepatocellular carcinoma, and brain tumors showed promising results and confirmed preclinical data [22–25]. In particular, low background uptake in the brain, lung, and bowel was observed. Other PET agents targeting the system x_C_^−^ transporter, [^18^F]hGTS13 and [^18^F]FASu, have also been recently described in preclinical models [26–28].

Preclinical research has recently shown that increased system x_C_^−^ activity enhances cancer cell dependency on glucose and a previously unappreciated role of system x_C_^−^ was uncovered [29, 30]. This demonstrates that both the glycolytic and the glutathione pathways are connected. Limiting glucose supply with inhibitors of glucose transporters can selectively kill cancer cells with high levels of system x_C_^−^ or suppress tumor growth. This may further assist with future therapeutic strategies to target the metabolic vulnerability in tumors with high system x_C_^−^ expression.

The work presented here is the initial evaluation of [^18^F]FSPG in patients with head and neck cancer (HNC), colorectal cancer (CRC), or non-Hodgkin lymphoma (NHL). The selection of these malignancies was based upon the tracer’s favorable performance in preclinical studies. For example, strong tumor uptake was demonstrated in subcutaneous human NCI-HT29 colon tumor models [16]. Moreover, xCT-targeted therapy has shown potential use for arresting tumor growth and/or sensitizing these cancer cells, reemphasizing the transporter’s role in disease pathogenesis [31–34]. The physiologic biodistribution of [^18^F]FDG was also considered for this selection of malignancies, as high [^18^F]FDG accumulation in the brain and bowel inevitably limits the contrast for tumor imaging in these regions. Due to the pilot nature of this investigation with [^18^F]FSPG, imaging with [^18^F]FDG was an inclusion criterion and was used for general comparison.

The purpose of this study was twofold: (1) to provide preliminary data regarding the pattern of [^18^F]FSPG PET uptake in patients with HNC, CRC, or NHL and (2) to compare these profiles with [^18^F]FDG PET, thus exploring possible clinical applications for this radiotracer in these disease entities.

## Methods

The protocol for this study was reviewed and approved by the U.S. Food and Drug Administration (eIND 108509), the Institutional Review Board at Stanford University, and the Scientific Review Committee at the Stanford Cancer Institute. Fifteen subjects with histologically confirmed, newly diagnosed, or recurrent head and neck cancer (HNC, *n* = 5), colorectal cancer (CRC, *n* = 5), or non-Hodgkin lymphoma (NHL, *n* = 5) were recruited (ClinicalTrials.gov Identifier: NCT01186601). Detailed inclusion and exclusion criteria are listed in the Supplementary data section. To be eligible, each subject was required to have a positive whole-body [^18^F]FDG scan beforehand. The average time between the [^18^F]FDG scan and the [^18^F]FSPG scan was 10 ± 6 days. [^18^F]FDG PET/CT studies were done as a standard-of-care clinical scan, approximately 60 (± 15) min after a standard radioactive dose of 555 MBq ± 20% (15 mCi ± 20%) was administered. A low-dose, non-contrast CT was performed for attenuation correction and anatomic localization for all scans [35, 36].

Prior to the [^18^F]FSPG PET/CT scan, a brief physical exam was performed, and vital signs, blood, and urine samples were collected. [^18^F]FSPG was administered as a slow intravenous bolus injection over 60 s. The mean ± standard deviation of the radioactive dose given was 301.4 ± 28.1 MBq (8.1 ± 0.8 mCi), with a range of 270.1–336.7 MBq (7.3–9.1 mCi). After each respective tracer injection, the cannula and injection system were flushed with 10 mL normal saline (0.9% NaCl).

Three [^18^F]FSPG PET/CTs were then acquired sequentially to capture different time points after tracer injection for evaluation of temporal change in biodistribution. The images were obtained using a GE Discovery PET/CT scanner (either model D600 or D690). The first image acquisition, with a total duration of 45 min, was performed immediately after the injection of tracer. It was performed as five sequential whole-body (vertex to mid-thigh) PET scans after obtaining one CT (140 kV, range 10–85 mAs) for attenuation correction and anatomic localization. Each of the 5 PET scans gradually increased in the number of minutes per bed position as follows: 30 s/bed, 30 s/bed, 1 min/bed, 2 min/bed, and 2 min/bed. The second and third whole-body PET/CT scans, each with a duration of approximately 30 min (3 min/bed position), were started at 60 and 105 min post-tracer injection, respectively. The patients were asked to void before the second and third scan session to reduce their radiation exposure and to improve visualization of the pelvic structures.

After the scans were completed, and again the next day, additional sets of ECG, vital signs, and blood and urine samples were obtained. Any adverse events either noted by the participant or the research team were recorded. Seven days later, the patient was contacted by phone to determine if there were any interim adverse events or medication changes.

[^18^F]FSPG and [^18^F]FDG PET images were analyzed as described previously [23].

### Statistical analysis

The Mann–Whitney *U* test was applied to compare [^18^F]FDG and [^18^F]FSPG SUV tumor values and SUV tumor-to-background ratios at 60 min across different tumor indications. *P* values < 0.05 were considered as statistically significant.

## Results

**Fig. 1:**
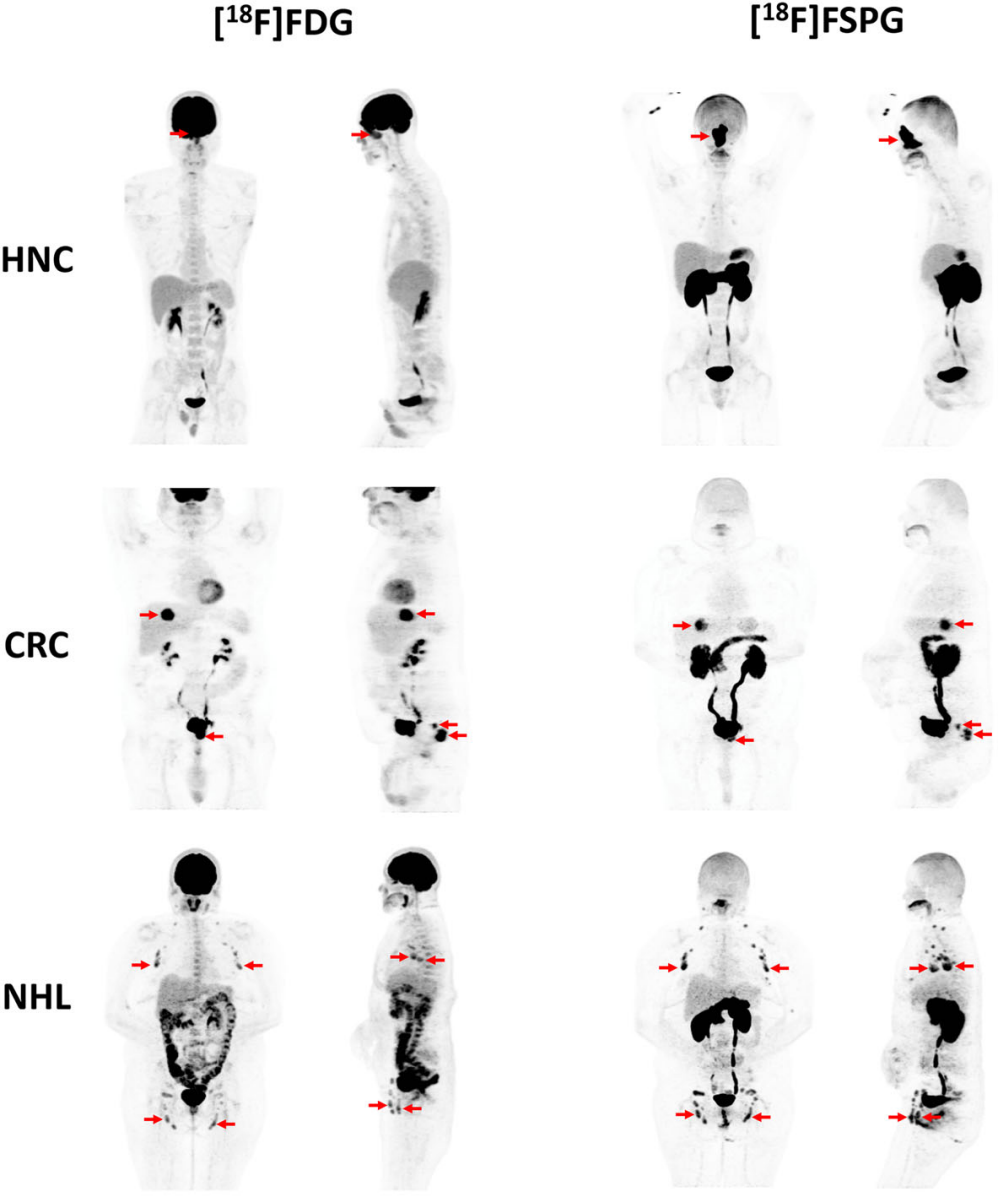
Whole-body maximum intensity projection (MIP) images are shown of a representative participant with HNC, CRC, or NHL. The [^18^F]FDG PET images (front and side views) are shown on the left, and the [^18^F]FSPG PET images (front and side views) are shown on the right. Red arrows indicate sites of malignancy on each scan

Twelve men and three women, with an average age of 66 years (range 45–87), were recruited. All five subjects with HNC had squamous cell carcinoma (SCC), and all CRCs were adenocarcinomas. The subjects with NHL had a variety of subtypes including diffuse large B cell (*n* = 1), follicular (*n* = 1), cutaneous T cell (*n* = 1), and mantle cell (*n* = 2). The full patient demographics are given in Table 1. Whole-body maximum intensity projection (MIP) images are shown in Fig. 1 of a representative participant with HNC, CRC, or NHL.

**Table 1:** Demographic information for the 15 human participants imaged with [^18^F]FSPG PET

Patient	Gender	Age	Pathology	Prior treatment (months before)
1	Male	44	HNC, SCC; nasal cavity	None
2	Male	73	HNC, SCC; recurrent (oropharynx-oral cavity)	Chemoradiation, laryngectomy (156)
3	Male	71	HNC, SCC; left maxillary sinus	Chemoradiation (3.5)
4	Male	74	HNC, SCC; larynx	None
5	Male	72	HNC, SCC; nasopharynx	None
6	Male	62	CRC, adenocarcinoma	Hemicolectomy (36), chemotherapy (6)
7	Male	64	CRC, adenocarcinoma	Hemicolectomy (16), chemotherapy (9)
8	Male	69	CRC, adenocarcinoma	Chemoradiation (6)
9	Female	50	CRC, adenocarcinoma	Chemotherapy neoadjuvant and rectosigmoid resection (26), chemotherapy (17)
10	Male	69	CRC, adenocarcinoma	None
11	Male	72	NHL, DLBCL	Chemoradiation (10), chemotherapy (0.5)
12	Female	78	NHL, mantle cell	None
13	Male	65	NHL, mantle cell	Chemotherapy (5)
14	Male	87	NHL, follicular	None
15	Female	43	NHL, cutaneous T-cell	None

No adverse events were observed, either in terms of self-described symptoms or clinically relevant deviations in their vital signs or laboratory values. As observed before in prior studies [21], visual analysis of the PET images showed consistent physiologic biodistribution of [^18^F]FSPG across the subjects, with prominent diffuse activity throughout the pancreas (average SUV at 60 min was 7.2 ± 1.5). There was additional low-grade and variable activity in the oral cavity and oropharynx, salivary glands, thyroid gland, mediastinal blood pool, and liver. Some subjects also showed mild diffuse activity in the stomach. The tracer showed predominant clearance through the kidneys and excretion into the bladder. Beyond this, there was very low background activity in the brain, chest, abdomen, and pelvis. Time-activity-curve analysis for all seven time points per subject showed rapid clearance of the radiotracer from the blood pool (Supplementary Figure 1). More specific results separated by cancer type are presented next.

### Head and neck cancer

**Fig. 2:**
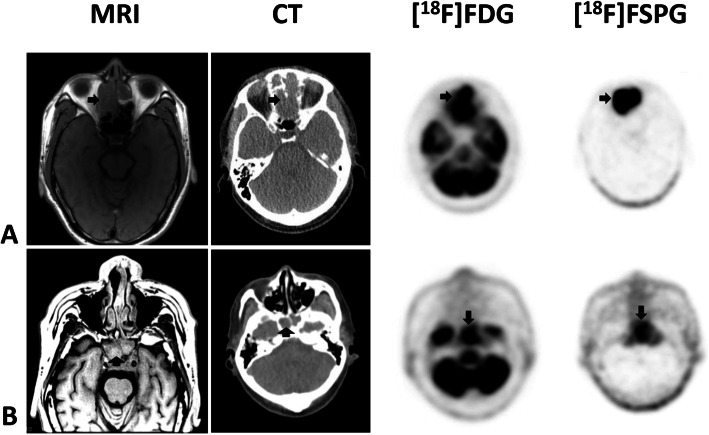
Combined image set shows two representative subjects with primary head and neck cancer (HNC). Shown from left to right are the axial T1-weighted MRI, CT, [^18^F]FDG PET, and [^18^F]FSPG PET images. The first subject (row **a**) has a large nasal mass while the second subject (row **b**) has a mass in the clivus/nasopharynx. The black arrows indicate the lesions. Any unmarked uptake on the PET scans is physiologic. All modalities show the lesions, although MRI and PET are clearer than CT. The relative uptake of [^18^F]FSPG and [^18^F]FDG within all 5 HNC subjects is similar (SUV of 2.76 ± 2.13 for [^18^F]FSPG and 4.92 ± 2.16 for [^18^F]FDG), although the complete lack of [^18^F]FSPG background uptake in normal brain (SUV of 0.1) versus [^18^F]FDG (SUV of 7.88 ± 1.45) makes evaluation of the [^18^F]FSPG images much easier and more suitable, for example, in radiation treatment planning

**Fig. 3:**
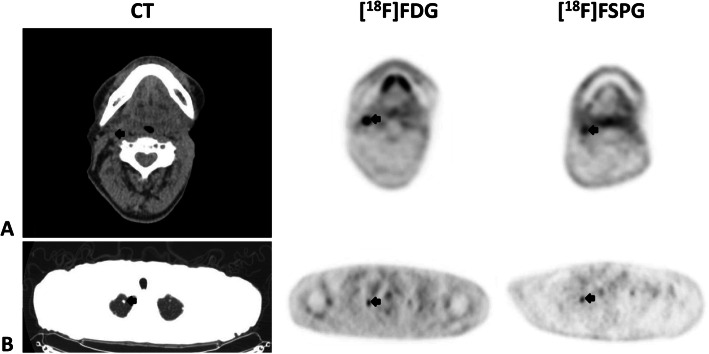
Combined image set shows two representative subjects with metastatic head and neck cancer (HNC). Shown from left to right are the axial CT (upper is soft-tissue window, lower is lung window), [^**18**^F]FDG PET, and [^**18**^F]FSPG PET images. The first subject (row **a**) has a right level 2 cervical lymph node, while the second subject (row **b**) has multiple pulmonary nodules of which a right apical nodule is shown. The black arrows indicate the lesions. Any unmarked uptake on the PET scans is physiologic. Of note, both the cervical node and the pulmonary nodule are subcentimeter in size (9 mm for the cervical node (**a**) and 7 mm for the right upper lobe pulmonary nodule (**b**)). The lesions are seen on all modalities, and both the [^**18**^F]FDG and [^**18**^F]FSPG PET images have uptake in both the cervical node (SUV of 8.7 and 2.6, respectively) and the pulmonary nodule (SUV of 2 and 1.5, respectively)

Of the five subjects with head and neck squamous cell cancer, four were newly diagnosed, and one had recurrent disease. Each had disease involvement of different regions including the nasal cavity, left tongue base, left maxillary sinus, and larynx (patients 1–5, Table 1). Figure 2 shows a combination image of two of these subjects highlighting the primary tumor as seen on MRI, CT, [^18^F]FDG PET, and [^18^F]FSPG PET. In these two examples, the tumor is evident on all modalities. In comparison to the [^18^F]FDG PET scan, the lack of any background brain activity in the [^18^F]FSPG scans is of particular note. Figure 3 shows examples of regional nodal metastases and distant lung metastases as seen on CT, [^18^F]FDG PET, and [^18^F]FSPG PET.

Time-activity-curve analysis for [^18^F]FSPG was performed for all 7 scans per subject (except subject 1 who had only three scans). The mean activity in both the primary and metastatic tumor lesions remained relatively constant throughout the duration of imaging (Supplementary Figure 2A). The activity in the primary tumor is higher than blood pool activity from 15 min post-injection (p.i.) onwards, and the activity in the metastases is higher than blood pool activity at 40 min p.i. onwards. Supplementary Figure 2A also highlights again the very low background brain activity throughout the duration of imaging. At 60 min p.i., the mean lesion SUV for [^18^F]FSPG was significantly lower than [^18^F]FDG for both the primary (2.8 ± 2.1 versus 4.9 ± 2.2) and metastatic (1.3 ± 0.7 versus 3.0 ± 1.4) lesions (Table 2), whereas the tumor-to-background values using various reference organs including the brain, mediastinal blood pool, gluteal muscle, and liver tended to be higher for [^18^F]FSPG compared to [^18^F]FDG.

**Table 2:** Average SUV_mean_ values obtained at 60 min post-injection of [^18^F]FSPG and [^18^F]FDG for each of the cancer types

	Head and neck cancer			Colorectal cancer			Non-Hodgkin lymphoma		
SUV 60 min p.i. (g/ml)	[^18^F]FSPG	[^18^F]FDG	Ratio SUV_**FSPG**_/SUV_**FDG**_	[^18^F]FSPG	[^18^F]FDG	Ratio SUV_**FSPG**_/SUV_**FDG**_	[^18^F]FSPG	[^18^F]FDG	Ratio SUV_**FSPG**_/SUV_**FDG**_
	**(mean ± SD)**			**(mean ± SD)**			**(mean ± SD)**		
**Primary**	2.8 ± 2.1	4.9 ± 2.2	0.57	5.4 ± 1.3	6.9 ± 0.8	0.78	1.9 ± 3.1	1.9 ± 1.6	1.00
**Metastases**	1.3 ± 0.7	3.0 ± 1.4	0.43	2.5 ± 2.4	3.5 ± 3.5	0.71	-	-	
**Aorta**	0.8 ± 0.3	1.6 ± 0.3	0.50	0.9 ± 0.4	1.9 ± 0.5	0.47	1.0 ± 0.3	1.7 ± 0.3	0.59
**Pancreas**	7.4 ± 1.4	1.7 ± 0.2	4.35	7.0 ± 1.4	1.7 ± 0.4	4.12	7.1 ± 1.7	1.9 ± 0.3	3.74
**Heart**	0.7 ± 0.1	2.4 ± 1.3	0.29	0.8 ± 0.4	3.9 ± 1.5	0.21	0.9 ± 0.1	2.0 ± 0.5	0.45
**Kidney whole**	9.8 ± 1.3	3.2 ± 0.6	3.06	16.1 ± 7.3	4.7 ± 2.6	3.43	11.4 ± 2.7	4.4 ± 1.4	2.59
**Kidney cortex**	8.2 ± 0.6	2.6 ± 0.1	3.15	11.3 ± 3.7	2.8 ± 1.0	4.04	10.6 ± 2.8	2.7 ± 0.4	3.93
**Brain**	0.1 ± 0.0	7.9 ± 1.4	0.01	0.12 ± 0.04	7.8 ± 2.0	0.01	0.1 ± 0.0	8.2 ± 3.6	0.01
**Muscle**	0.2 ± 0.04	0.7 ± 0.1	0.29	0.2 ± 0.1	0.6 ± 0.1	0.33	0.3 ± 0.1	0.8 ± 0.1	0.38
**Lung**	0.2 ± 0.04	0.4 ± 0.1	0.50	0.3 ± 0.1	0.5 ± 0.2	0.60	0.3 ± 0.1	0.5 ± 0.2	0.60
**Liver**	1.2 ± 0.3	2.3 ± 0.2	0.52	1.5 ± 0.4	2.3 ± 0.3	0.65	1.5 ± 0.1	2.6 ± 0.3	0.58
**Tumor: brain**	27.6	0.6	46.00	45.3	0.9	50.33	18.9	0.2	94.50
**Tumor: aorta**	3.3	3.0	1.10	5.7	3.7	1.54	1.9	1.1	1.73
**Tumor: muscle**	12.6	7.2	1.75	20.9	11.2	1.87	6.7	2.5	2.68
**Tumor: liver**	2.3	2.2	1.05	3.7	3.0	1.23	1.3	0.8	1.63
**Tumor: pancreas**	0.4	2.9	0.14	0.8	1.7	0.47	0.3	1	0.30
**Tumor: kidney**	0.3	1.5	0.20	3.4	1.5	2.27	0.2	0.4	0.50

The tumor-to-background ratios are also shown and were taken with respect to six different relevant backgrounds: brain, aorta (blood pool), muscle (left gluteal muscle), liver, pancreas, and kidney

### Colorectal cancer

**Fig. 4:**
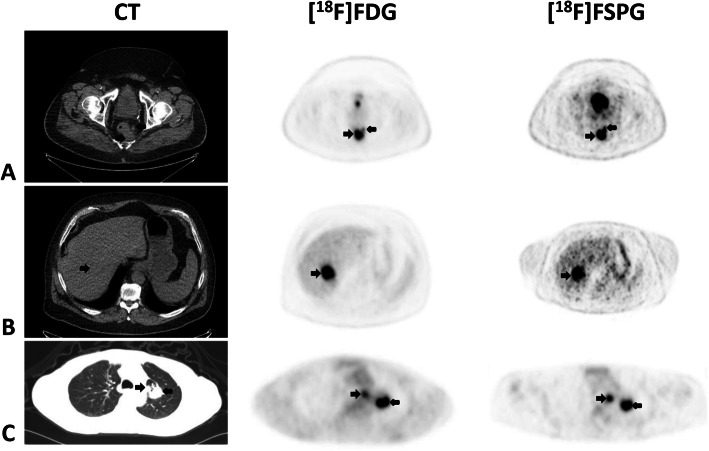
Combined image set shows three representative subjects with metastatic colorectal cancer (CRC). Displayed from left to right are the axial CT (upper two rows are soft-tissue window, lowest row is lung window), [^18^F]FDG PET, and [^18^F]FSPG PET images. The first subject (row **a**) has a rectal mass and an adjacent small perirectal lymph node, the second subject (row **b**) has a liver metastasis, and the third subject (row **c**) has pulmonary metastases on the left and an ipsilateral mediastinal lymph node metastasis. The black arrows indicate the lesions. Any unmarked uptake on the PET scans is physiologic. The lesions are seen on all modalities, and both the [^18^F]FDG and [^18^F]FSPG PET images have similar uptake across all lesions (SUV rectal mass (**a**) 15.8 and 12, liver lesion (**b**) 17.4 and 16.1, and pulmonary metastasis (**c**) 7.4 and 8.3, for [^18^F]FDG and [^18^F]FSPG, respectively)

All five subjects had recurrent metastatic adenocarcinoma (patients 6–10, Table 1). Sites of metastases include the lungs, liver, and regional lymph nodes. Figure 4 shows a combination image of three of these subjects highlighting the primary tumor and metastases as seen on CT, [^18^F]FDG PET, and [^18^F]FSPG PET. In these examples, the tumor is evident on all modalities, although one of the liver lesions was not well evaluated due to lack of intravenous contrast on CT. However, both the [^18^F]FDG and [^18^F]FSPG PET scans showed the lesions well.

Time-activity-curve analysis for [^18^F]FSPG was done for all 7 scans per subject. The mean activity in the primary tumor continues to rise to a peak at 60 min p.i. and is above the mediastinal blood pool activity from 10 min p.i. onwards. The metastatic lesions demonstrate stable increased uptake throughout the duration of imaging and remain above blood pool activity from 15 min p.i. onwards (Supplementary Figure 2B). The background liver SUV_mean_ also demonstrates rapid washout. Liver background activity is below the primary lesion at 10 min p.i., below the metastatic lesions from the initial time point onwards, and above the aortic blood pool activity at 40 min p.i. At 60 min p.i., the mean lesion SUV for [^18^F]FSPG was lower than [^18^F]FDG for both the primary tumor (5.4 ± 1.3 versus 6.9 ± 0.8) and metastases (2.5 ± 2.4 versus 3.5 ± 3.5) (Table 2). However, the tumor-to-background ratios for [^18^F]FSPG were significantly higher than [^18^F]FDG values with gluteal muscle as background.

### Non-Hodgkin lymphoma

**Fig. 5:**
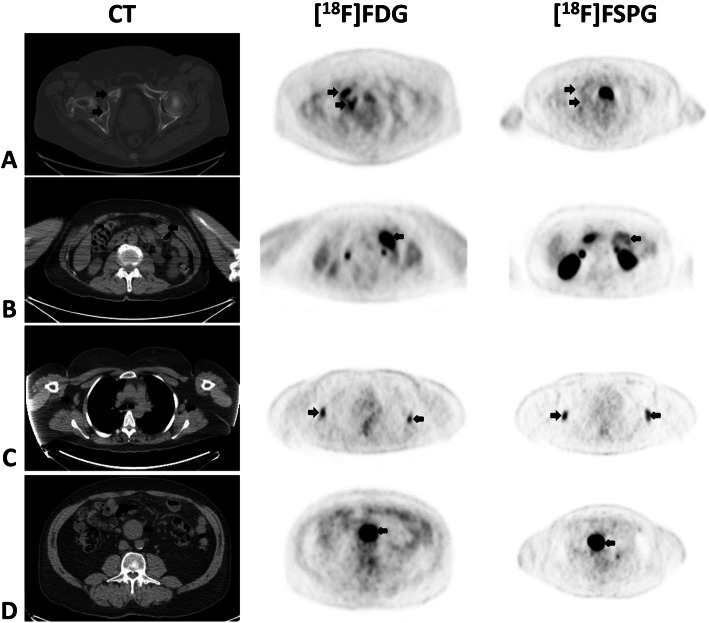
Combined image set shows four representative subjects with non-Hodgkin lymphoma (NHL). Displayed from left to right are the axial CT (soft-tissue window shown at various cranio-caudal levels), [^18^F]FDG PET, and [^18^F]FSPG PET images. The first subject (row **a**) with diffuse large B cell lymphoma has a large soft-tissue mass in the right hip invading the bone, the second subject (row **b**) with follicular lymphoma has a large mesenteric lymph node on the left, the third subject (row **c**) with mantle cell lymphoma has small bilateral axillary lymph nodes, and subject 4 (row **d**) with mantle cell lymphoma has a large central mesenteric mass. The black arrows indicate the lesions. Any unmarked uptake on the PET scans is physiologic. The lesions are seen on all modalities, although the [^18^F]FSPG PET scan shows very little uptake for some of the lesions (SUV right hip mass (**a**) 11.3 and 2.9, left mesenteric lymph node (**b**) 9.2 and 3.2, right axillary lymph node (**c**) 3.5 and 1.1, and central mesenteric mass (**d**) 11.1 and 21.9, for [^18^F]FDG and [^18^F]FSPG, respectively)

Different subtypes of NHL were represented in this cohort including one with diffuse large B cell, one with follicular, one with cutaneous T cell, and two with mantle cell lymphoma. Some had localized (stage I) disease while several others had diffuse (stage IV) disease, including bone marrow involvement. None had pulmonary or splenic involvement. Across all the subjects, sites of nodal involvement include cervical, axillary, mediastinal, retroperitoneal, mesenteric, pelvic, and inguinal stations. Figure 5 shows a combination image of four of these subjects highlighting some of these lesions as seen on CT, [^18^F]FDG PET, and [^18^F]FSPG PET. In these examples, the tumor is evident on most modalities, but with some variability. In particular, depending on the subtype of NHL, the nodal lesions, which are all evident on the [^18^F]FDG scans, demonstrate very little to very high uptake with [^18^F]FSPG.

The nodal lesions show stable increased uptake throughout the duration of imaging and uptake is above blood pool activity from 30 min p.i. onwards (Supplementary Figure 2C). At 60 min p.i., the mean lesion SUV for [^18^F]FSPG was comparable to [^18^F]FDG (1.9 ± 3.1 versus 1.9 ± 1.6), but with greater variability (Table 2), and the tumor-to-background values using gluteal muscle were significantly higher for [^18^F]FSPG than for [^18^F]FDG.

### Per-lesion analysis

**Fig. 6:**
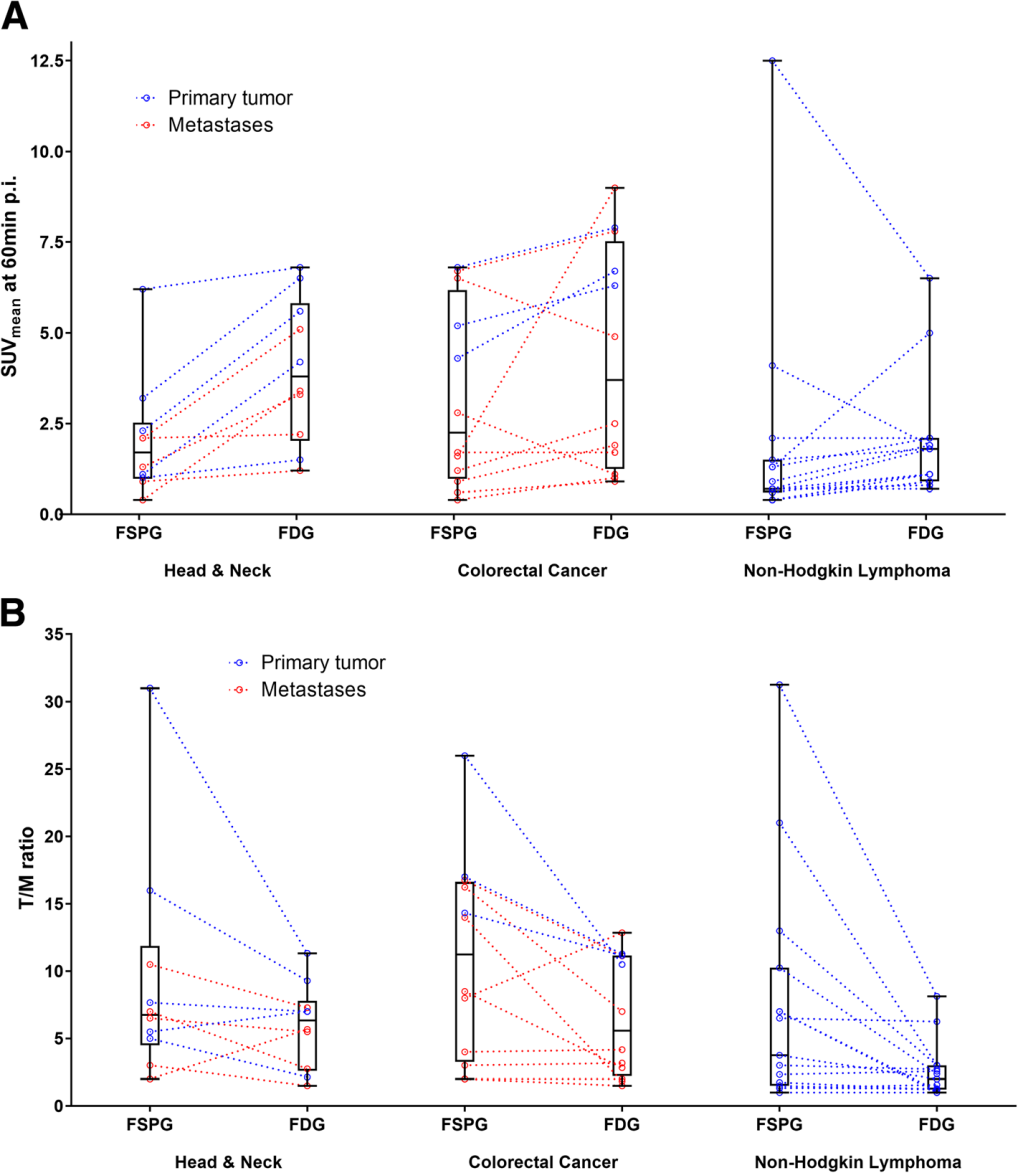
**a** Box plots of the SUV values for [^18^F]FSPG and [^18^F]FDG at 60 min post-injection (p.i.). The actual SUV values of the individual lesions from the primary tumor are shown as blue dots, and the individual metastatic lesions are shown as red dots. Corresponding [^18^F]FDG and [^18^F]FSPG lesion uptake is shown by connecting lines. **b** Box plots of the tumor-to-muscle (T/M) ratio for [^18^F]FSPG and [^18^F]FDG lesion uptake at 60 min p.i.

A direct comparison of [^18^F]FSPG and [^18^F]FDG lesion uptake for each tumor indication at 60 min p.i. is given in Fig. 6a with further detailed sub-analysis for each individual primary tumor and metastatic lesion. In general, those lesions that showed high uptake with one tracer also showed high uptake with the other tracer with some exceptions. [^18^F]FSPG showed lower uptake than [^18^F]FDG in the majority of head and neck and colorectal cancer cases, whereas the two tracers showed comparable uptake in most non-Hodgkin lymphoma lesions. Figure 6b shows the corresponding tumor-to-muscle ratios. Higher tumor-to-background ratios for [^18^F]FSPG support the notion of at least comparable lesion detection in these three indications despite the lower absolute uptake in HNC and CRC lesions.

## Discussion

A consistent physiologic biodistribution pattern for [^18^F]FSPG was found in patients with HNC, CRC, or NHL. The subjects with HNC all showed uptake in tumor lesions with both [^18^F]FSPG and [^18^F]FDG PET, although there were some key differences between these scans. Most notably, the lack of any [^18^F]FSPG background uptake in the normal brain allows for easier interpretation of [^18^F]FSPG PET scans, especially for skull base lesions close to the brain (Fig. 2). This includes one subject with a large nasal mass and another with a nasopharyngeal mass extending to the clivus. Evaluation of regional nodal and distant pulmonary metastases was visually indistinguishable between the two radiopharmaceuticals, although several of these lesions were quite small (subcentimeter in size). SUV analysis showed that the primary tumors had a higher SUV value on the [^18^F]FSPG PET scan (by approximately 1.0 SUV more) in comparison to the metastases. The absolute [^18^F]FSPG SUV values for these lesions were also lower than for the comparative [^18^F]FDG PET scan, although the tumor-to-background ratios were higher. The only exception was a patient with recurrent SCC of the left tongue base. In that particular case, the [^18^F]FDG PET scan had a notably higher uptake than the [^18^F]FSPG PET scan. Subsequent biopsy confirmed SCC recurrence. Incidentally, this subject was given antibiotic therapy for a presumed oral infection for 1 week between his [^18^F]FDG and [^18^F]FSPG PET scans. As such, the [^18^F]FDG PET signal presumably reflects both the tumor and the associated infection and may also have been lower if done after the antibiotic therapy. [^18^F]FSPG uptake has been associated with an active inflammatory disease state by measuring xCT activity in activated M1 macrophages [19], but its role in inflammatory or infectious lesions remains to be explored in more detail.

Epstein-Barr virus (EBV) status was available for three patients. EBV-positive lesions showed lower SUV values on both scans in comparison to those that were EBV-negative. [^18^F]FDG PET functional parameters have previously been shown to be significantly associated with plasma EBV DNA load [37], and the roles of both have been demonstrated for prognostication in HNC [38]. As a core biomarker in the setting of tumorigenesis, the possibility of [^18^F]FSPG also correlating with EBV would be noteworthy.

All subjects with CRC showed uptake on both [^18^F]FSPG and [^18^F]FDG PET scans. In these subjects, all of whom had recurrent, metastatic adenocarcinoma, both radiopharmaceuticals visually performed quite similarly with no major discrepancies noted in terms of lesion detection. SUV analysis shows a very similar pattern to the HNC cases where the absolute SUV values for the primary and metastatic lesions were lower for the [^18^F]FSPG PET scan than in comparison to the [^18^F]FDG PET scan, but with higher tumor-to-background levels. Overall, these findings indicate that both radiopharmaceuticals were similarly effective for detecting CRC in this small patient cohort. K-ras mutation status was also available for three patients with thoracic metastases. Mutated k-ras has been shown to increase [^18^F]FDG uptake, possibly by upregulation of GLUT1 [39, 40]. The pulmonary nodule positive for k-ras mutation showed higher uptake with [^18^F]FDG than the wildtype (3.5 versus 1.9), whereas the [^18^F]FSPG SUV values were comparable (1.2 versus 1.0), which may allude to the different pathways targeted by the two tracers.

The subjects with NHL showed the greatest amount of variability between the [^18^F]FSPG and [^18^F]FDG PET scans. In fact, not all the subjects with NHL showed significant uptake above background with [^18^F]FSPG PET. One subject with diffuse large B cell lymphoma and another with follicular lymphoma both had uptake levels essentially equivalent to the liver background. Both of these subjects showed mild-to-moderate uptake with [^18^F]FDG. Another subject with cutaneous T cell lymphoma showed very similar mildly increased uptake in comparison to [^18^F]FDG. The most interesting subtype of NHL was mantle cell lymphoma, of which there were two subjects. The first showed the lowest SUV values of all 5 subjects with NHL, while the other showed the highest. In fact, the latter subject’s [^18^F]FSPG SUV values were nearly double that of the comparative [^18^F]FDG scan although both scans were visually intensely active. As a marker of the proliferative index, the Ki-67 staining for the first subject with mantle cell lymphoma was 6% while for the second subject was 20%. The SUV of [^18^F]FDG rises with increased proliferative activity and biological aggressiveness of the tumor tissue. SUV was shown to have a statistically significant positive correlation with the proliferative index Ki-67 across a variety of subtypes of non-Hodgkin’s lymphoma [41]. This may explain some of the discrepancy between their [^18^F]FSPG and [^18^F]FDG scans. Moreover, the lesions from the indolent follicular and cutaneous T cell lymphomas had lower [^18^F]FSPG SUV and uptake ratios than the more aggressive types (mean SUV of 3.4 versus 5.4; mean uptake ratio of 1.7 versus 3.0). Counterintuitive to the consensus in the literature which supports lower [^18^F]FDG uptake in indolent lymphomas as well [42], the corresponding [^18^F]FDG values were comparable between the two groups (mean SUV of 5.2 versus 5.0; mean uptake ratio of 1.9 versus 1.9).

As in other [^18^F]FSPG PET studies, the scalp showed incidental prominent physiologic uptake, possibly corresponding to the x_C_^−^ transporter’s role in hair pigmentation [43]. Intense diffuse activity was also noted throughout the pancreas on [^18^F]FSPG PET, which would limit evaluation of primary pancreatic cancers. However, [^18^F]FSPG PET has been recently studied in patients with metastasized pancreatic ductal adenocarcinoma with promising results [44]. Due to prominent radioactive clearance through the kidneys and urinary bladder, evaluation of these regions is additionally difficult, although concomitant diuretic administration was not utilized.

The results of this pilot study are encouraging but have limitations. Foremost is the small sample size for each of the cancer indications associated with the preliminary nature of this project. However, the goal was not to definitively characterize the behavior of [^18^F]FSPG for each of these cancer indications, but rather to understand whether there is any uptake or role at all for this radiopharmaceutical in each cancer type. Indeed, the cancer types chosen were based on known increased activity and the proven role by [^18^F]FDG. It is noteworthy that this also poses a strong inherent potential bias against [^18^F]FSPG due to an underestimation of the added clinical impact in non-FDG-avid disease. The goal was not to outperform [^18^F]FDG in these indications, but to learn about the potential utility of [^18^F]FSPG in these indications while addressing a different metabolic pathway for imaging. In this light, the results of this study show that [^18^F]FSPG indeed performed well for all these cancer types except for certain subtypes of lymphoma. However, since only 1–2 subjects per type were imaged, results should be cautiously evaluated. Overall, the SUV for [^18^F]FSPG was generally similar but slightly lower than [^18^F]FDG across all 15 subjects, and when evaluated as a ratio relative to background uptake, it was slightly higher than [^18^F]FDG. Statistically significant differences were observed in some instances (absolute uptake in HNC and the tumor-to-muscle ratios in CRC and NHL) but should be interpreted with caution since, again, the small sample sizes limit the power for each of these evaluations.

It is generally well accepted that system x_C_^−^ mediated uptake of cystine and glutathione biosynthesis have pro-survival functions under stress conditions. The unexpected and unique observation made by Koppula et al. [29] on the pro-cell death function of system x_C_^−^ in the context of glucose starvation is of special interest. It was reported from preclinical investigations that tumor cells with high system x_C_^−^ activity would have a more limited metabolic flexibility and more reliance on glucose for survival than those with low system x_C_^−^ activity. High intracellular levels of cystine from increased system x_C_^−^ activity can be potentially toxic, if the constitutive reduction to the more soluble cysteine is limited. Constant replenishing of the cellular NADPH pool is required and renders such cells dependent on the pentose phosphate pathway and high glycolytic activity [30]. This may also explain the high concordance between [^18^F]FDG and [^18^F]FSPG in this study. Another publication reports about similar observations on reduced nutrient flexibility upon increased system x_C_^−^ expression [45]. Accordingly, tumors with high [^18^F]FSPG uptake could represent those that would be more vulnerable than those with low [^18^F]FSPG uptake when glucose is limited. More research is needed to verify the model for the proposed role of system x_C_^−^ on glucose dependence to better understand the possible implications of these observations.

These initial promising results with [^18^F]FSPG warrant further evaluation in a larger cohort of cancer patients to confirm these preliminary findings. Moreover, it would be beneficial to investigate the possible role of [^18^F]FSPG PET in imaging [^18^F]FDG non-avid disease and assessing therapy response. Additional information on the metabolic phenotype and adaptations of tumors against oxidative stress may provide a better understanding of the underlying tumor biology and chemoresistance mechanisms that can potentially be useful for therapy selection and monitoring with [^18^F]FSPG PET.

## Conclusion

[^18^F]FSPG is a promising PET radiopharmaceutical that specifically targets the x_C_^−^ transporter. Avid uptake of [^18^F]FSPG was observed in subjects with HNC and CRC, and variable uptake was seen in subjects with NHL. Overall, the results are encouraging and show concordant visualization with [^18^F]FDG PET across 15 subjects with 3 different cancer types. Future studies based on larger numbers of subjects and those with a wider array of primary and recurrent or metastatic tumors are planned to further evaluate the utility of this novel radiopharmaceutical.

## Supplementary information

**Additional file 1: Supplementary Table 1.** Specific inclusion and exclusion criteria for enrollment of the study participants in the [^18^F]FSPG PET/CT imaging trial.

**Additional file 2: Supplementary Figure 1.** Time-activity curves for [^18^F]FSPG across all 15 subjects showing the mean blood pool activity as a marker for clearance of the tracer from the blood pool. For each cancer type, the values are very similar and show rapid clearance from the blood pool with low tracer levels remaining at 60 minutes post-injection. The vertical bars represent the standard deviation of the data, but are shown only for the HNC subjects for ease of visibility.

**Additional file 3: Supplementary Figure 2.** Time-activity curves across all 7 imaging time-points for all subjects for each of the 3 cancer indications, head and neck cancer (A), colorectal cancer (B), and non-Hodgkin lymphoma (C). The values shown are mean ± standard deviation. In each case, the primary tumor and metastases are shown, as well as the blood pool (aorta) and other background regions as appropriate (i.e., brain for head and neck cancer, and liver for colorectal cancer).

## Data Availability

The datasets supporting the conclusions of this article are included within the article.

## Abbreviations

[^18^F]FSPG: (4*S*)-4-(3-[^18^F]Fluoropropyl)-l-glutamate
PET/CT: Positron emission tomography/computed tomography
HNC: Head and neck cancer
CRC: Colorectal cancer
NHL: Non-Hodgkin lymphoma
[^18^F]FDG: ^18^F-fluoro-2-deoxyglucose
SUV: Standardized uptake value
SCC: Squamous cell carcinoma
MRI: Magnetic resonance imaging
p.i.: Post-injection
EBV: Epstein-Barr virus

## References

[1] Vander Heiden MG, Cantley LC, Thompson CB (2009). Understanding the Warburg effect: the metabolic requirements of cell proliferation. Science..

[2] Vander Heiden MG, DeBerardinis RJ (2017). Understanding the intersections between metabolism and cancer biology. Cell..

[3] Hensley CT, Wasti AT, DeBerardinis RJ (2013). Glutamine and cancer: cell biology, physiology, and clinical opportunities. J Clin Invest.

[4] Zhu L, Ploessl K, Zhou R, Mankoff D, Kung HF (2017). Metabolic imaging of glutamine in cancer. J Nucl Med.

[5] Lewerenz J, Hewett SJ, Huang Y (2013). The cystine/glutamate antiporter system x(c)(-) in health and disease: from molecular mechanisms to novel therapeutic opportunities. Antioxid Redox Signal.

[6] Arensman MD, Yang XS, Leahy DM (2019). Cystine-glutamate antiporter xCT deficiency suppresses tumor growth while preserving antitumor immunity. Proc Natl Acad Sci U S A.

[7] Badgley MA, Kremer DM, Maurer HC (2020). Cysteine depletion induces pancreatic tumor ferroptosis in mice. Science..

[8] Podoloff DA, Ball DW, Ben-Josef E (2009). NCCN task force: clinical utility of PET in a variety of tumor types. J Natl Compr Cancer Netw.

[9] Fletcher JW, Djulbegovic B, Soares HP (2008). Recommendations on the use of ^18^F-FDG PET in oncology. J Nucl Med.

[10] Bomanji JB, Costa DC, Ell PJ (2001). Clinical role of positron emission tomography in oncology. Lancet Oncol.

[11] Culverwell AD, Scarsbrook AF, Chowdhury FU (2011). False-positive uptake on 2-[^18^F]-fluoro-2-deoxy-D-glucose (FDG) positron-emission tomography/computed tomography (PET/CT) in oncological imaging. Clin Radiol.

[12] Metser U, Miller E, Lerman H, Even-Sapir E (2007). Benign nonphysiologic lesions with increased ^18^F-FDG uptake on PET/CT: characterization and incidence. AJR Am J Roentgenol.

[13] Shreve PD, Anzai Y, Wahl RL (1999). Pitfalls in oncologic diagnosis with FDG PET imaging: physiologic and benign variants. Radiographics..

[14] Farwell MD, Pryma DA, Mankoff DA (2014). PET/CT imaging in cancer: current applications and future directions. Cancer..

[15] Fathinul F, Nordin AJ, Lau WF (2013). [^18^F]FDG-PET/CT is a useful molecular marker in evaluating tumour aggressiveness: a revised understanding of an in-vivo FDG-PET imaging that alludes the alteration of cancer biology. Cell Biochem Biophys.

[16] Koglin N, Mueller A, Berndt M (2011). Specific PET imaging of xC- transporter activity using a ^18^F-labeled glutamate derivative reveals a dominant pathway in tumor metabolism. Clin Cancer Res.

[17] Greenwood HE, McCormick PN, Gendron T (2019). Measurement of tumor antioxidant capacity and prediction of chemotherapy resistance in preclinical models of ovarian cancer by positron emission tomography. Clin Cancer Res.

[18] McCormick PN, Greenwood HE, Glaser M (2019). Assessment of tumor redox status through (S)-4-(3-[^18^F]fluoropropyl)-L-glutamic acid PET imaging of system xc(-) activity. Cancer Res.

[19] Chae SY, Choi CM, Shim TS (2016). Exploratory clinical investigation of (4S)-4-(3-^18^F-fluoropropyl)-l-glutamate PET of inflammatory and infectious lesions. J Nucl Med.

[20] Smolarz K, Krause BJ, Graner FP (2013). (S)-4-(3-^18^F-fluoropropyl)-L-glutamic acid: an ^18^F-labeled tumor-specific probe for PET/CT imaging--dosimetry. J Nucl Med.

[21] Mosci C, Kumar M, Smolarz K, et al. Characterization of physiologic ^18^F FSPG uptake in healthy volunteers. Radiology. 2016;142000. 10.1148/radiol.2015142000 26785040

[22] Baek S, Choi CM, Ahn SH (2012). Exploratory clinical trial of (4S)-4-(3-[^18^F]fluoropropyl)-L-glutamate for imaging xC- transporter using positron emission tomography in patients with non-small cell lung or breast cancer. Clin Cancer Res.

[23] Mittra ES, Koglin N, Mosci C (2016). Pilot preclinical and clinical evaluation of (4S)-4-(3-[^18^F]fluoropropyl)-L-glutamate (^18^F-FSPG) for PET/CT imaging of intracranial malignancies. PLoS One.

[24] Kavanaugh G, Williams J, Morris AS (2016). Utility of [^18^F]FSPG PET to image hepatocellular carcinoma: first clinical evaluation in a US population. Mol Imaging Biol.

[25] Baek S, Mueller A, Lim YS (2013). (4S)-4-(3-^18^F-fluoropropyl)-L-glutamate for imaging of xC transporter activity in hepatocellular carcinoma using PET: preclinical and exploratory clinical studies. J Nucl Med.

[26] Beinat C, Gowrishankar G, Shen B (2019). The characterization of ^18^F-hGTS13 for molecular imaging of xC (-) transporter activity with PET. J Nucl Med.

[27] Webster JM, Morton CA, Johnson BF (2014). Functional imaging of oxidative stress with a novel PET imaging agent, ^18^F-5-fluoro-L-aminosuberic acid. J Nucl Med.

[28] Yang H, Jenni S, Colovic M (2017). ^18^F-5-Fluoroaminosuberic acid as a potential tracer to gauge oxidative stress in breast cancer models. J Nucl Med.

[29] Koppula P, Zhang Y, Shi J, Li W, Gan B (2017). The glutamate/cystine antiporter SLC7A11/xCT enhances cancer cell dependency on glucose by exporting glutamate. J Biol Chem.

[30] Liu X, Olszewski K, Zhang Y (2020). Cystine transporter regulation of pentose phosphate pathway dependency and disulfide stress exposes a targetable metabolic vulnerability in cancer. Nat Cell Biol.

[31] Yoshikawa M, Tsuchihashi K, Ishimoto T (2013). xCT inhibition depletes CD44v-expressing tumor cells that are resistant to EGFR-targeted therapy in head and neck squamous cell carcinoma. Cancer Res.

[32] Ma MZ, Chen G, Wang P (2015). Xc- inhibitor sulfasalazine sensitizes colorectal cancer to cisplatin by a GSH-dependent mechanism. Cancer Lett.

[33] Gout PW, Buckley AR, Simms CR, Bruchovsky N (2001). Sulfasalazine, a potent suppressor of lymphoma growth by inhibition of the x(c)- cystine transporter: a new action for an old drug. Leukemia..

[34] Lo M, Wang YZ, Gout PW (2008). The x(c)- cystine/glutamate antiporter: a potential target for therapy of cancer and other diseases. J Cell Physiol.

[35] Iagaru A, Kundu R, Jadvar H, Nagle D (2009). Evaluation by ^18^F-FDG-PET of patients with anal squamous cell carcinoma. Hell J Nucl Med.

[36] Iagaru AH, Mittra ES, McDougall IR, Quon A, Gambhir SS (2008). ^18^F-FDG PET/CT evaluation of patients with ovarian carcinoma. Nucl Med Commun.

[37] Chang KP, Tsang NM, Liao CT (2012). Prognostic significance of ^18^F-FDG PET parameters and plasma Epstein-Barr virus DNA load in patients with nasopharyngeal carcinoma. J Nucl Med.

[38] Shen T, Tang LQ, Luo DH (2015). Different prognostic values of plasma Epstein-Barr virus DNA and maximal standardized uptake value of ^18^F-FDG PET/CT for nasopharyngeal carcinoma patients with recurrence. PLoS One.

[39] Kawada K, Nakamoto Y, Kawada M (2012). Relationship between ^18^F-fluorodeoxyglucose accumulation and KRAS/BRAF mutations in colorectal cancer. Clin Cancer Res.

[40] Iwamoto M, Kawada K, Nakamoto Y (2014). Regulation of ^18^F-FDG accumulation in colorectal cancer cells with mutated KRAS. J Nucl Med.

[41] Papajík T, Mysliveček M, Sedová Z (2011). Standardised uptake value of ^18^F-FDG on staging PET/CT in newly diagnosed patients with different subtypes of non-Hodgkin’s lymphoma. Eur J Haematol.

[42] Rodriguez M, Rehn S, Ahlström H, Sundström C, Glimelius B (1995). Predicting malignancy grade with PET in non-Hodgkin’s lymphoma. J Nucl Med.

[43] Chintala S, Li W, Lamoreux ML (2005). Slc7a11 gene controls production of pheomelanin pigment and proliferation of cultured cells. Proc Natl Acad Sci U S A.

[44] Cheng MF, Huang YY, Ho BY (2019). Prospective comparison of (4S)-4-(3-^18^F-fluoropropyl)-L-glutamate versus ^18^F-fluorodeoxyglucose PET/CT for detecting metastases from pancreatic ductal adenocarcinoma: a proof-of-concept study. Eur J Nucl Med Mol Imaging.

[45] Shin CS, Mishra P, Watrous JD (2017). The glutamate/cystine xCT antiporter antagonizes glutamine metabolism and reduces nutrient flexibility. Nat Commun.

