# Supplementary material for: Initial evaluation of (4S)-4-(3-[18F]fluoropropyl)-l-glutamate (FSPG) PET/CT imaging in patients with head and neck cancer, colorectal cancer, or non-Hodgkin lymphoma
Source: EJNMMI Res. 2020 Aug 28;10:100. doi: 10.1186/s13550-020-00678-2 (PMC7455665; doi:10.1186/s13550-020-00678-2)
Supplement: Supplementary file 1 — Additional file 1: Supplementary Table 1. Specific inclusion and exclusion criteria for enrollment of the study participants in the [18F]FSPG PET/CT imaging trial. [file 13550_2020_678_MOESM1_ESM.docx]

| **Inclusion Criteria** | **Exclusion Criteria** |
| --- | --- |
| - Written informed consent - Males/females ≥18 years - Females of no childbearing potential or females of childbearing potential but not pregnant or nursing. - Patient had an [^18^F]FDG PET/CT in clinical routine that still showed tumor mass with high certainty, and primary cancer disease is histologically confirmed. - The [^18^F]FDG PET/CT above was performed within 4 weeks prior to [^18^F]FSPG PET/CT. - Adequate recovery (excluding alopecia) from previous surgery, radiation, and chemotherapy - ECOG (Eastern Cooperative Oncology Group) performance status of 0-2, determined within one week prior to treatment with [^18^F]FSPG. - Confirmation of adequate function of major organs and systems. - No clinically relevant deviations in renal function as determined by Cockcroft and Gault method using serum creatinine at screening. - No malfunction equivalent to CTC (Common toxicity criteria, CTCAE v3.0) toxicities grade > 2 of the liver (ALAT; bilirubin). - No therapy or biopsy between [^18^F]FDG PET/CT and [^18^F]FSPG PET/CT. - Life expectancy of at least 3 months. | - Concurrent severe and/or uncontrolled and/or unstable medical disease other than cancer or inflammation - Known sensitivity to the study drug or components of the preparation - Alcohol or drug dependence |
