# Supplementary figures and images for: Initial evaluation of (4S)-4-(3-[18F]fluoropropyl)-l-glutamate (FSPG) PET/CT imaging in patients with head and neck cancer, colorectal cancer, or non-Hodgkin lymphoma
Source: EJNMMI Res. 2020 Aug 28;10:100. doi: 10.1186/s13550-020-00678-2 (PMC7455665; doi:10.1186/s13550-020-00678-2)

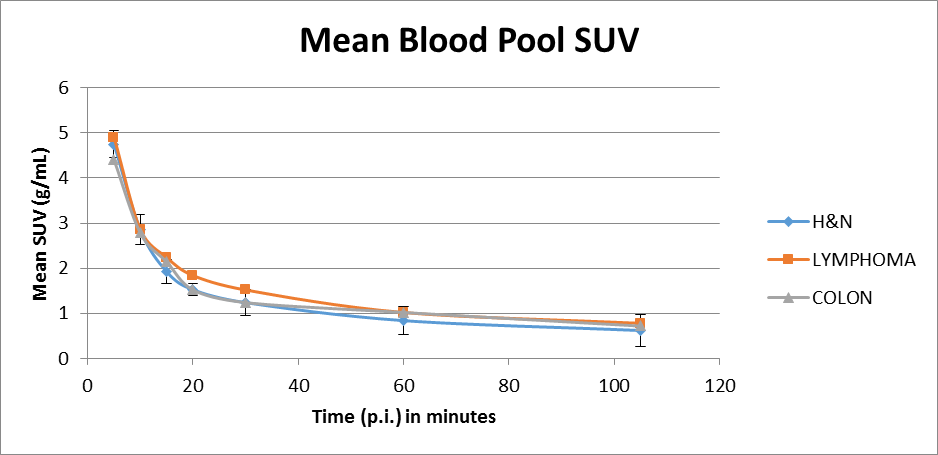

Supplement: Supplementary file 2 — Additional file 2: Supplementary Figure 1. Time-activity curves for [18F]FSPG across all 15 subjects showing the mean blood pool activity as a marker for clearance of the tracer from the blood pool. For each cancer type, the values are very similar and show rapid clearance from the blood pool with low tracer levels remaining at 60 minutes post-injection. The vertical bars represent the standard deviation of the data, but are shown only for the HNC subjects for ease of visibility. [file 13550_2020_678_MOESM2_ESM.tif]

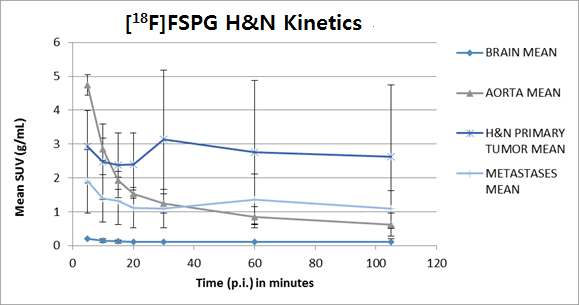

Supplement: Supplementary file 3 — Additional file 3: Supplementary Figure 2. Time-activity curves across all 7 imaging time-points for all subjects for each of the 3 cancer indications, head and neck cancer (A), colorectal cancer (B), and non-Hodgkin lymphoma (C). The values shown are mean ± standard deviation. In each case, the primary tumor and metastases are shown, as well as the blood pool (aorta) and other background regions as appropriate (i.e., brain for head and neck cancer, and liver for colorectal cancer). [file 13550_2020_678_MOESM3_ESM.zip › Supp Figure 2A.tif]

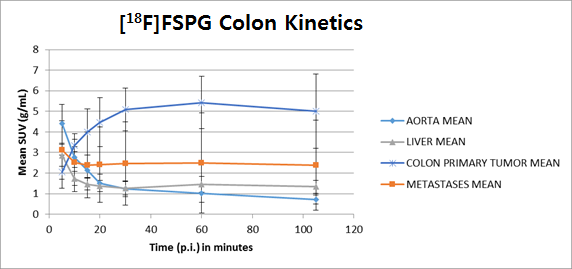

Supplement: Supplementary file 3 — Additional file 3: Supplementary Figure 2. Time-activity curves across all 7 imaging time-points for all subjects for each of the 3 cancer indications, head and neck cancer (A), colorectal cancer (B), and non-Hodgkin lymphoma (C). The values shown are mean ± standard deviation. In each case, the primary tumor and metastases are shown, as well as the blood pool (aorta) and other background regions as appropriate (i.e., brain for head and neck cancer, and liver for colorectal cancer). [file 13550_2020_678_MOESM3_ESM.zip › Supp Figure 2B.tif]

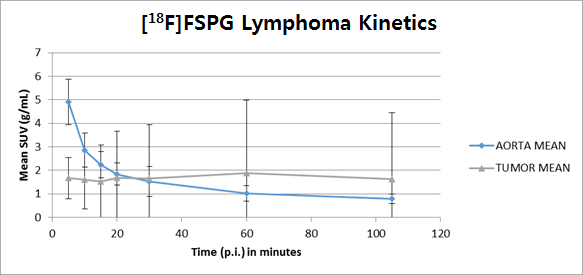

Supplement: Supplementary file 3 — Additional file 3: Supplementary Figure 2. Time-activity curves across all 7 imaging time-points for all subjects for each of the 3 cancer indications, head and neck cancer (A), colorectal cancer (B), and non-Hodgkin lymphoma (C). The values shown are mean ± standard deviation. In each case, the primary tumor and metastases are shown, as well as the blood pool (aorta) and other background regions as appropriate (i.e., brain for head and neck cancer, and liver for colorectal cancer). [file 13550_2020_678_MOESM3_ESM.zip › Supp Figure 2C.tif]
